# Cardiovascular-kidney-metabolic Syndrome Entangled: “In Rhythm with Time, from Days to Years”

**DOI:** 10.31662/jmaj.2024-0291

**Published:** 2024-11-18

**Authors:** Atsushi Mizuno

**Affiliations:** 1 Department of Cardiovascular Medicine, St. Luke’s International Hospital, Tokyo, Japan

**Keywords:** sacubitril/valsartan, heart failure, cardiovascular-kidney-metabolic syndrome, diabetes, HbA1c

Recently, sodium-glucose cotransporter-2 (SGLT2) inhibitors, that were initially developed as antidiabetic agents, have been found to prevent kidney failure and demonstrate remarkable cardioprotective effects, particularly in reducing heart failure-related hospitalizations and cardiovascular mortality. Additionally, glucagon-like peptide 1 (GLP-1) receptor agonists (GLP-1RAs) not only improve insulin resistance and blood glucose levels but also reduce body weight and significantly lower cardiovascular mortality. These discoveries have led to the emergence of a new concept: cardiovascular-kidney-metabolic (CKM) syndrome ^(1)^. CKM syndrome is characterized by pathophysiological interactions between metabolic risk factors, chronic kidney disease (CKD), and the cardiovascular system, leading to multiorgan dysfunction and a high incidence of adverse cardiovascular events. CKM syndrome includes health disorders related to the interconnections between obesity, diabetes, CKD, and cardiovascular disease (CVD), encompassing conditions including heart failure, atrial fibrillation, coronary artery disease, stroke, and peripheral artery disease.

The mechanism underlying CKM syndrome remain to be explored; however, the established efficacy of SGLT2 inhibitors and GLP-1RAs in preventing cardiovascular events emphasizes the critical importance of managing diabetes as a key underlying condition. These antidiabetic agents offer a clear example of how diabetes medications can have a profound influence on cardiovascular outcomes, solidifying their role in addressing the interconnected nature of CKM syndrome. Interestingly, the present topic explores the potential impact of sacubitril/valsartan―a medication primarily used to treat heart failure and hypertension―on diabetes, particularly on HbA1c levels, providing a reverse perspective on this relationship. Watanabe et al. analyzed data from the REVIEW-HF registry, which included 470 adult patients, 39% of whom had diabetes, and evaluated changes in HbA1c following sacubitril/valsartan administration ^(2)^. The results demonstrated a decrease in HbA1c levels after 3 months of treatment, with the effect sustained for up to 12 months. This is noteworthy because sacubitril/valsartan is not traditionally classified as an antidiabetic drug, yet it may have a meaningful influence on key diabetic indicators. This exploration of a nondiabetic drug affecting diabetes-related variables represents a novel angle within the broad CKM concept.

Although the exact mechanisms behind sacubitril/valsartan’s effect on HbA1c are unclear, it is hypothesized that the inhibition of neprilysin results in increased levels of natriuretic peptides and decreased degradation of GLP-1, leading to improved insulin sensitivity and lower blood glucose levels. The ability of sacubitril/valsartan to slow the progression of heart failure may also contribute to improved glycemic control. These findings were first noted in the subanalyses of the landmark PARADIGM-HF and PARAGON-HF trials ^(3)^, which demonstrated the efficacy of sacubitril/valsartan in patients with heart failure. However, a substantial distinction between those trials and the current study by Watanabe et al. is the mean HbA1c levels observed. In PARADIGM-HF and PARAGON-HF, the mean HbA1c was 7.4%, whereas, in the REVIEW-HF study, the mean HbA1c in a predominantly Asian cohort―60% of whom did not have diabetes―was 6.4%. This lower baseline HbA1c makes the findings in this population particularly intriguing. Similarly, data from Korea also indicated a reduction in HbA1c levels in patients with diabetes following sacubitril/valsartan treatment, although a rebound was observed after 18 months. However, it should be noted that this study is not an intervention trial; therefore, although it supports previous findings, it cannot definitively establish causality between sacubitril/valsartan and HbA1c reduction. Nevertheless, this study offers valuable insights that align with existing knowledge, particularly from a broad internal medicine perspective ^(4)^.

From this macroviewpoint, it becomes clear that the treatment landscape for heart failure is evolving, with increasing consideration given to the relationship among diabetes, obesity, and CVD. As the management of chronic conditions becomes complex, the need for interdisciplinary expertise grows. Clinicians must stay informed about accumulating evidence and understand the broad context in which these findings are situated. In this regard, the evolving role of antidiabetic agents and traditional cardiovascular medications, such as sacubitril/valsartan, highlights the importance of continuous evidence review.

An often-overlooked aspect in studies on HbA1c and cardiovascular treatments is the role of seasonal variability ^(5)^. Notably, HbA1c control tends to worsen in winter, a trend that has been observed globally. In the Korean study, the lack of precise annual data collection may have contributed to the observed HbA1c rebound. By contrast, this study appears to have more controlled enrollment and minimal temporal variability, leading to a stable and homogeneous dataset. This suggests that the stability of HbA1c results may have been influenced by the study’s methodology, which accounted for potential seasonal fluctuations.

In the future, studies evaluating the impact of sacubitril/valsartan on HbA1c should continue to account for temporal and seasonal variations in patient data. Additionally, consideration of other macrolevel factors, such as circadian variability, the impact of holidays, and even differences in prescribing practices among healthcare providers, could offer new insights. Although the effect of sacubitril/valsartan on HbA1c is becoming clear, caution is warranted in making definitive conclusions without considering these broad influences. Future research should incorporate such variability to better understand the drug’s overall impact.

In conclusion, the potential for sacubitril/valsartan to influence HbA1c adds another dimension to the CKM syndrome framework, highlighting the complex and interconnected nature of chronic diseases such as heart failure, diabetes, and CKD. As more data is collated, it will become increasingly clear how cardiovascular and metabolic systems are intricately intertwined across different timescales, highlighting the importance of comprehensive strategies for managing CKM syndrome. By continuing to build on this growing body of evidence, clinicians can make more informed decisions that improve patient outcomes across multiple domains.

## Article Information

### Conflicts of Interest

None

### Disclaimer

Atsushi Mizuno is one of the Editors of JMA Journal and on the journal’s Editorial Staff. He was not involved in the editorial evaluation or decision to accept this article for publication at all.
